# Supplementary material for: Hybrid Models and Biological Model Reduction with PyDSTool
Source: PLoS Comput Biol. 2012 Aug 9;8(8):e1002628. doi: 10.1371/journal.pcbi.1002628 (PMC3415397; doi:10.1371/journal.pcbi.1002628)
Supplement: Text S4 — Complete source code for the PyDSTool package (version 0.88.120504). Includes API documentation and help files linking to web pages. This file is identical to the current public release on Sourceforge.net. (ZIP) [file pcbi.1002628.s004.zip › PyDSTool/html/PyDSTool.Toolbox.optimizers.step.gradient_step-pysrc.html]

xml version="1.0" encoding="ascii"?


PyDSTool.Toolbox.optimizers.step.gradient\_step


| Home | Trees | Indices | Help | PyDSTool |
| --- | --- | --- | --- | --- |

Package PyDSTool ::
Package Toolbox ::
Package optimizers ::
Package step ::
Module gradient\_step

[hide private]

[frames] | no frames]

# Source Code for Module PyDSTool.Toolbox.optimizers.step.gradient\_step

```
 1   
 2  # Matthieu Brucher 
 3  # Last Change : 2007-08-10 23:15 
 4   
 5  """ 
 6  Computes a gradient step for a specific function at a specific point 
 7  """ 
 8   


9 -class GradientStep(object):


10    """ 
11    The simple gradient step 
12    """ 


13 -  def __call__(self, function, point, state):


14      """ 
15      Computes a gradient step based on a function and a point 
16      """ 
17      gradient = function.gradient(point) 
18      state['gradient'] = gradient 
19      step = - gradient 
20      state['direction'] = step 
21      return step

22
```

  


| Home | Trees | Indices | Help | PyDSTool |
| --- | --- | --- | --- | --- |

|  |  |
| --- | --- |
| Generated by Epydoc 3.0.1 on Fri May 4 15:24:23 2012 | http://epydoc.sourceforge.net |
